# Enhancing Hyperheuristics for the Knapsack Problem through Fuzzy Logic

**DOI:** 10.1155/2021/8834324

**Published:** 2021-01-25

**Authors:** Frumen Olivas, Ivan Amaya, José Carlos Ortiz-Bayliss, Santiago E. Conant-Pablos, Hugo Terashima-Marín

**Affiliations:** Tecnologico de Monterrey, Escuela de Ingeniería y Ciencias Ave, Eugenio Garza Sada 2501 Sur Col. Tecnológico C.P. 64849, Monterrey, Nuevo Leon, Mexico

## Abstract

Hyperheuristics rise as powerful techniques that get good results in less computational time than exact methods like dynamic programming or branch and bound. These exact methods promise the global best solution, but with a high computational time. In this matter, hyperheuristics do not promise the global best solution, but they promise a good solution in a lot less computational time. On the contrary, fuzzy logic provides the tools to model complex problems in a more natural way. With this in mind, this paper proposes a fuzzy hyperheuristic approach, which is a combination of a fuzzy inference system with a selection hyperheuristic. The fuzzy system needs the optimization of its fuzzy rules due to the lack of expert knowledge; indeed, traditional hyperheuristics also need an optimization of their rules. The fuzzy rules are optimized by genetic algorithms, and for the rules of the traditional methods, we use particle swarm optimization. The genetic algorithm will also reduce the number of fuzzy rules, in order to find the best minimal fuzzy rules, whereas traditional methods already use very few rules. Experimental results show the advantage of using our approach instead of a traditional selection hyperheuristic in 3200 instances of the 0/1 knapsack problem.

## 1. Introduction

Hyperheuristics are high-level methods created to solve problems by either selecting among different solvers [1] or by generating new ones based on the components of others [2]. These solvers are usually referred to as low-level heuristics (or simply, heuristics). Since heuristics are approximation methods, they have the advantage of being fast to execute, but they cannot guarantee to find the optimal solution. Then, hyperheuristics attempt to choose the best heuristic for each type of problem to improve the quality of the solutions. Burke et al. [3] refer to hyperheuristics as “high-level heuristics,” and classify them into two broad categories: selection and generation. In this work, we focus on selection hyperheuristics. As the name suggests, selection hyperheuristics decide which is the best heuristic to apply in different states of the problem. In general, one heuristic is likely to be better than the others for some specific instances. This phenomenon causes that selection hyperheuristics to choose this heuristic most of the time—perhaps more than needed. Throughout fuzzy logic, we think we can overcome this problem by creating a more balanced way in which the hyperheuristics select heuristics. Another mechanism of the hyperheuristics that can be enhanced is how they select the heuristics, and this is usually done by the difference between the current state of the problem (represented by a vector of metrics or features) and a set of rules generated by a search and optimization method. A fuzzy inference system has fuzzy rules that can determine which heuristic to select through the defuzzification process, which involves the use of autogenerated knowledge or the knowledge of an expert.

Fuzzy logic comprises a series of tools [4, 5] designed to model a problem in the way humans would do it. For instance, when we talk about the weather, we ignore the exact temperature, but we can communicate if it is hot or cold (and others will understand). Similarly, we can decide what to do according to inaccurate measurements. A fuzzy inference system (FIS) is a rule-based expert system that processes inputs with the help of a knowledge base (fuzzy rules) and brings the result as the output. In this regard, Mamdani [6] proposed the first type of FIS, and Sugeno [7] proposed a faster way to compute the output of a FIS. The main difference between these two types of FIS is how they compute the output. While Mamdani uses a more natural manner to model a problem by a combination of fuzzy sets, Sugeno uses equations for a faster calculation of the output. In this investigation, we have preferred Sugeno as FIS for its faster computational time.

The knapsack problem is still a difficult combinatorial problem to solve for exact methods [8], so in this research, we adopt it as a benchmark for comparison, to see if our fuzzy approach can get better results when compared to nonfuzzy selection hyperheuristics. The difficulty in this problem lies in having to select a subset of items *s*_*i*_⊆*S*_*I*_, ∀*i* ∈ *I*, that does not exceed the total weight *w* ≤ *W* allowed by the knapsack and maximizes a profit *P*=∑_*i*=1_^*n*^*p*_*i*_*x*_*i*_.

In our proposed approach, we use a FIS as a selection hyperheuristic. Under our proposal, the problem features are inputs to the FIS, while the output is the selected heuristic. Then, the defuzzification process is how the hyperheuristic computes the “distance” between rules and the current problem state. In other words, we change the way the hypeheuristic works internally by incorporating a FIS. For the sake of brevity, we will refer to this type of hyperheuristic as a fuzzy hyperheuristic.

The main innovations derived from this research are as follows. First, we have proposed a model that optimizes the fuzzy rules of a FIS and uses it as a hyperheuristic. We optimize the model using easy-to-solve instances and apply it to hard-to-solve instances, without reoptimizing its rules. These tests prove, with the help of a genetic algorithm (GA), that our model gets the knowledge to solve a knapsack problem and save it as fuzzy rules. This model combines the advantages of selection hyperheuristics and fuzzy logic inference to increase the quality of the results, compared to traditional selection hyperheuristics optimized by particle swarm optimization (PSO). The optimization performed by the GA has the aim of getting the consequents of the fuzzy rules and also reducing the number of rules, while the optimization of the traditional methods performed by PSO is by finding the values of the rules and their consequents, but these rules are less (4, 6 or 8) than the fuzzy rules (128), that is why the GA also reduces the number of fuzzy rules. For the optimization process of both methods, we use a set of 120 instances of the knapsack problem and 680 instances of the same type for testing, and we also include 2400 hard instances for testing. Even when the methods were not optimized with the hard instances (which are of a special type of instances), the results on these instances show that our proposed fuzzy approach has an advantage over the traditional hyperheuristic methods.

We organize the rest of the paper as follows.  Section 2 captures the state-of-the-art works related to this research.  Section 3 presents the theoretical basis and concepts used in this research.  Section 4 explains the methodology used to optimize and transform a FIS into a hyperheuristic.  Section 5 presents all the experiments, and the results we got.  Section 6 discusses the results in a graphical view and a statistical comparison. Finally, in Section 7, we present the conclusions and feature work.

## 2. State-of-the-Art

This section covers the main works conducted on related topics like selection and generation hyperheuristics, metaheuristics to combinatorial problems, fuzzy logic, knapsack problem, genetic algorithms to optimize fuzzy inference systems, and the combination of fuzzy logic with hyperheuristics.

Ross [1] described some ideas related to hyperheuristics and several applications to different problems like Boolean satisfaction, scheduling, and bin packing. Cowling et al. [9] presented a hyperheuristic that can adapt to the problem under exploration. They use a choice function that determines the best heuristic on each state of the problem, leading to an autonomous hyperheuristic which automatically adapts its parameters and gets superior results when compared to other techniques. Sosa-Ascencio et al. [10] proposed an algorithm that uses genetic programming to generate selection hyperheuristics, using a grammar extracted from the existing heuristics. Their generated heuristics have an improved performance when compared against human-designed heuristics, but not in all instances. Lopez-Camacho et al. [11] created a framework to develop and test hyperheuristics to solve bin packing problem (BPP) instances in 1D and 2D, by considering items with regular and irregular forms in the case of 2D, and proposed an evolutionary selection and constructive hyperheuristics that solve efficiently distinct types of problems. Terashima-Marin et al. [12] proposed a generalized hyperheuristic for solving BPPs with different forms, which include regular (rectangular) and irregular (concave or convex) ones. Using a variable-length genetic algorithm, they evolve hyperheuristics that solve the problems more efficiently than the best single low-level heuristic for each instance. Tsai et al. [13] proposed a hyperheuristic scheduling algorithm to find better solutions for a cloud computing system, and their results show a significant reduction of the makespan compared with several state-of-the-art algorithms using two detection operators to automatically determine when to change the low-level heuristic and a perturbation operator to fine-tune the solutions got. Sabar et al. [14] used a grammatical evolution hyperheuristic to evolve several heuristic components to produce a generic problem solver. They also included an adaptive memory mechanism for the hyperheuristic. Their results show the advantage of their approach when compared to some bespoke methods from literature, and this is because of the use of an adaptive memory mechanism, which contains a collection of the best solutions. Ortiz-Bayliss et al. [15] applied a genetic algorithm (GA) with variable-length chromosomes to evolve selection hyperheuristics applied to real and synthetic constraint satisfaction problems, using seven heuristics. Their results confirm the robustness of their approach in unseen instances without loss of efficiency. Koulinas et al. [16] used particle swarm optimization (PSO) to optimize a hyperheuristic applied to solve the resource-constrained project scheduling problem. Their results are competitive against other approaches from the literature. Also, they claim a linear increase in computational time using their approach to solve a set of schedules. Ortiz-Bayliss et al. [2] proposed a method to generate new heuristics by evolving a set of features from other heuristics from the literature, applied to randomly generate constraint satisfaction problems, where they get better heuristics than the ones taken from literature, but could not achieve a general heuristic able to solve different types of problems. Maashi et al. [17] described a multiobjective hyperheuristic with a learning selection choice function. They used multiobjective evolutionary algorithms as low-level heuristics applied to the vehicle crashworthiness design problem. The results show the advantage of the hyperheuristic over the low-level heuristics on its own and also over an adaptive multimethod search from the literature. Zhao et al. [18] use an evolutionary hyperheuristic for location-routing problem with simultaneous pickup delivery where they use various metaheuristic techniques to guide the search and help the hyperheuristic to select the best low-level heuristic. Their proposal got better results than the best fine-tuned bespoke state-of-the-art approaches in the literature. Wang et al. [19] also applied a hyperheuristic to the location-routing problem but with the constraint of fuel consumption as a multiobjective problem where their results show better performance than the well-known NSGA-II algorithm. Yang et al. [20] use a hyperheuristic to select the best heuristic mutation operators from a pool to search for a solution in the space of the problem of dynamic economic and environmental load dispatch in order to determine the amount of electricity in a power plan, and their results show the effectiveness of their proposal. Soria et al. [21] use a hyperheuristic to select the most promising operator in the problems of vehicle routing and timetabling, their strategy is found a balance between diversification and intensification heuristics, and their results show an improved performance and new best-known solutions for the timetabling benchmark problem.

Zhao et al. [22] applied a discrete water wave optimization algorithm along with a greedy algorithm, and these methods have their advantages and disadvantage; to overcome their disadvantages, they use hybridization. The hybridization is a technique where, with the combination of two or more methods, their advantages are included in a single method. This is the fundamental idea of our proposal for this paper. Deng et al. [23] use a greedy algorithm with a population to tackle a job-shop scheduling problem where it does not allow waste time between two jobs: the greedy algorithm works with iterations and the population evolved by their destruction and construction. Their results show the advantage of using their proposal over other methods, and even they claim that their method could work in a real-world application. Ribas et al. [24] also use a greedy algorithm with a variable neighborhood search to a job-shop scheduling which has a constraint that increases the complexity of the problem by allowing a machine has a storage capability, so a machine can be not available before the previous machine has finished their job. They use two strategies to tackle this problem and to select the critical line which has the highest tardiness. Their comparison of results shows an improvement over other benchmark algorithms proposed for a problem related to their own problem, since they consider that their approach is the first in applying to this problem.

Zadeh [4] introduced the early concepts of fuzzy sets. For Zadeh, a fuzzy set is a class of objects with continuum grades of membership. Zadeh [5] also explained the main difference between classical logic and fuzzy logic. Besides, he presented problems classical logic cannot answer because of the uncertainty and imprecision of data, but fuzzy logic can, by using fuzzy sets and fuzzy rules. Martinez et al. [25] presented a comparison between the metaheuristics ant colony optimization (ACO) and a hierarchical genetic algorithm (HGA) for the optimization of fuzzy rules, using the idea of searching for the consequent of the rules and the activation/deactivation method. They concluded that ACO got better results than the HGA. Adanez et al. [26] used a GA to find the best antecedents of the fuzzy rules, while they used an identification method for the consequent. In their work, they used a Sugeno FIS that incorporates multidimensional membership functions, showing that these functions get better results than a single-dimensional membership function. Chhabra and Singh [27] proposed a Mamdani-type fuzzy model to tackle the estimation of cost and effort in the software development process, and they optimize their membership functions in the fuzzy model through a GA, getting better results than traditional methods. Lin [28] used a triangular fuzzy number to model the time needed for a machine to complete an assigned job in the flexible job-shop scheduling problem. Then, he applied a backtracking search algorithm to create hyperheuristics that schedule the jobs for each machine. The results and comparisons show the advantage of their proposal over state-of-the-art algorithms from the literature. Asmuni et al. [29] proposed a FIS as a metric for ordering the exams in a scholar calendar. They make the ordering through simple heuristics, but a FIS computes the complexity of the exams as a preprocessing step. The results over 12 benchmark problems datasets show that their approach produces excellent quality solutions. However, the computational time taken to optimize each fuzzy model is very significant. Asmuni et al. [30] extended their previous work [29], but this time for ordering courses instead of exams. They optimize their FIS as previously, by only moving some parameters of the membership functions. More particularly, they only optimize the parameters that cause an intersection between two membership functions. The results on 11 benchmark datasets show that their approach produces suitable quality solutions with low requirements for rescheduling. And same as the previous work, the only drawback is the time needed to optimize the fuzzy model. Chaudhuri and De [31] proposed a hybrid GA through hill-climbing methods applied to the timetabling of resources (teachers, classrooms, and students) from a university. They use a FIS to improve the objective function for the soft constraints, aside from the objective function from the GA to meet the hard constraints. They used hill climbing to improve the genetic operators within the GA. With an increase in computational complexity, their results show an advantage and achieve similarity better score over GA-based solutions. Jackson et al. [32] used a FIS to control the late acceptance parameter from the hyperheuristic, which makes a performance ranking of the applied heuristics, where they compare new evaluations with previous ones to see if there is an improvement. They do this not only with the last one but with a number *L* of previous solutions, and with this, the system decides which heuristic has the best performance. Their results show that the fuzzy control is effectively improving the performance of the hyperheuristic in seven of the twelve instances. An area of opportunity is the inclusion of other search state measures that help to avoid local minima or too much diversification of the search space leading to poor solutions. Zamli et al. [33] used a FIS as a selection hyperheuristic, with inputs using various metrics about the applied heuristics, and the system decides to “change,” “stay,” or “may change” the last heuristic. From their results, they found that the hyperheuristics are superior when compared to metaheuristics. Also, using a FIS to change the low-level heuristic applied, this shows an advantage when compared against most of the other strategies. Yang and Petrovic [34] proposed a new similarity metric between timetable problems represented as graphs, where nodes (vertex) are exams, and the edges are the number of students who will take the exam connected with the same edge. Each vertex has a weight that represents the number of students that will take that exam. They have a case base of problems, and they want to know the similarity of a new problem with the current ones. For this, they use different metrics, including one based on a FIS. They test the approach on real-world problems and comparing the results against state-of-the-art methods. The fuzzy similarity measure leads to an excellent selection of heuristics and outperforms the state-of-the-art solutions. They believe that their new fuzzy similarity measure can be applicable to other domains of problems.

Different approaches to tackle the knapsack problem have been proposed in the literature. Sahoo et al. [35] presented how the knapsack problem is related to the problem of optimal allocation of physical resource blocks. A generalization of the standard 0-1 knapsack problem as the set-union knapsack problem was proposed by Lin et al. [36]. Another representation of the knapsack problem is the joint radio communication, caching, and computing decision problem proposed by Dang and Peng [37]. Mengistu et al. [38] modeled the virtual machine placement problem in volunteer cloud computing as a bounded 0-1 multidimensional knapsack problem and developed three heuristic-based algorithms to meet the objectives and constraints specific to volunteer cloud computing. Some methods applied to solve the knapsack problem are a tissue *P* system with cell division [39] and a quantum-inspired binary wolf pack algorithm [40].

As a summary of the revised works, we believe that hyperheuristics have shown to be better than isolated heuristics for many situations and provide a reliable technique for solving problems where exact methods require an unfeasible computational time. We also mentioned different problems where hyperheuristics have excelled in performance and produce solutions close to optimal ones. Research on fuzzy logic provides the tools needed to model complex problems, which, combined with other methods, helps to get better results. The current literature still considers the knapsack problem a hard and exciting problem and contains plenty of other modern problems that can be formulated as this generic one. They also provide a variety of methods used to solve this problem, but no one method can solve all knapsack problems. We found evidence that supports that the optimization of a FIS through GAs is feasible. Then, we can use GAs to help the FIS improve the results. In the literature, there is a gap in the combination of fuzzy logic and hyperheuristics. The paper proposed by Zamli et al. [33] is the most related to our work since they also replace a selection hyperheuristic with a FIS (other works only use a FIS to solve an issue without replacing the hyperheuristic by the FIS). The main difference against our proposed approach is that their fuzzy hyperheuristic does not select the next heuristic to be applied and only keeps or changes the current heuristic applied. In our proposed approach, in every state of the problem, it takes into account the features of the problem to decide which is the best heuristic to select (same as hyperheuristics do).

## 3. Theoretical Basis and Problem Statement

### 3.1. Hyperheuristics

Heuristics are techniques created to solve specific problems faster or approximately due to the slowness of exact methods. Unfortunately, to get this advantage, heuristics sacrifice the assurance to get the global optimum. Hyperheuristics—usually considered as high-level heuristics [41]—represent a compelling option compared to a simple heuristic [1], which also creates a symphony between heuristics through the combination of these at certain times from resolving a problem, leading to getting (in most of the cases) better results than any of the heuristics in isolation. A hyperheuristic needs a set of rules to be optimized by some search and optimization methods to create this symphony of heuristics. Hyperheuristics can tackle the knapsack problem in the following manner. First, define the heuristics suitable for the problem; in our case, there are four of them. Second, select the features that characterize the problem state. Now, the hyperheuristic only needs a set of rules, which will help to select the best heuristic for each problem state. A search and optimization technique can find these rules. A set of rules for a hyperheuristic is a matrix where each row represents a rule. These rules contain information about the conditions where one heuristic should be preferred before the others. For example, in rule *R*_1_=[0.2, 0.4, 0.9, 3], the values 0.2, 0.4, and 0.9 correspond to features 1, 2, and 3, respectively. The last value in the rule indicates the index of the heuristic to apply; in this example, the rule will select the third heuristic. We can define the number of rules in the hyperheuristic arbitrarily:

(1) $$\begin{array}{c} {\text{HH}}_{\text{Rules}}=\left[\begin{array}{cccc} 0.2 & 0.4 & 0.9 & 3\\ 0.9 & 0.1 & 0.3 & 4\\ 0.5 & 0.8 & 0.9 & 1 \end{array}\right]. \end{array}$$

Suppose a hyperheuristic has the rules shown in equation (1) and assume the features from the current state of the problem are *F*=[0.8, 0.2, 0.4]. Given these conditions, the closest rule to the current problem state is the one from the second row (from the perspective of the Euclidean distance). So, in this case, the hyperheuristic will recommend using the fourth heuristic. We can create the rules of a hyperheuristic through the generation of a matrix with random values, in which the first *n* columns correspond to the *n* features and the last column corresponds to the heuristic for each rule from the heuristics available. To train (optimize) a hyperheuristic on a problem, we can use a search and optimization method to find the best values for the matrix.

### 3.2. Fuzzy Inference System

The Sugeno-type [7] FIS used in this investigation uses the defuzzification process of computing the weighted average of all rules weight (or firing strength) using equation (2), where *Rw*_*i*_ is the firing strength and *z*_*i*_ is the consequent of rule *i*. Our FIS has an output which contains all the heuristics to select, each of them as membership functions of constant type, i.e., the constant values 1, 2, 3, and 4 assigned to each of the four heuristics, respectively. From this perspective, one might think “wait, how can we combine two heuristics? and say the weighted average of (0.5)(1)+(0.5)(3)=2;” in other words, the half of heuristic 1 plus the half of heuristic 3 is heuristic 2. In fact, this is how our fuzzy hyperheuristic works, but the knowledge on what heuristics “combine” it will be optimized by a metaheuristic. Just to be clear, our proposed approach does not combine any heuristic, and this is an internal process of a Sugeno FIS; at the end, we are looking for the best fuzzy rules that select the best heuristic, given the features of a problem state:

(2) $$\begin{array}{c} \text{output}=\sum_{i=1}^{n}\frac{Rw_{i}z_{i}}{Rw_{i}}. \end{array}$$

### 3.3. Genetic Algorithms

**Figure 1:**
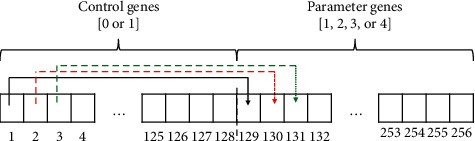
Representation of an individual as a chromosome. Control genes activate/deactivate the parameter genes.

The GA is a metaheuristic proposed by Holland [42], based on the natural evolution of species, while share their best genes to improve further generations. Using building blocks, GA can combine solutions to generate a better one. At the end (with enough generations), all individuals in a population converge towards the best solution. This is the method we used for the optimization of the fuzzy rules from our fuzzy approach. We use a custom discrete version of the original GA, where all genes can have only integer values. To optimize our fuzzy approach, we implement a custom two-point crossover method. All chromosomes have the configuration depicted in Figure 1, where the half are control genes and the other half are parameter genes. Control genes are used to manage the activation/deactivation of fuzzy rules, while parameter genes contain the consequent for each fuzzy rule.

**Figure 2:**
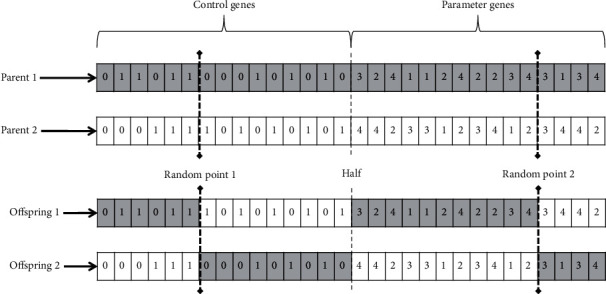
Example of a 2-point crossover between two chromosomes. A random point in the control genes and the other in the parameter genes, to force the combination of both sections of the chromosome.

In the selection process, individuals are chosen based on a tournament, where two individuals are randomly selected from the population (they may not be the same individual), the one with the best fitness is selected, stored, and removed from the population, the other individual is returned to the population ready to be selected in the next round, and in this way, until the necessary individuals are selected. For the crossover depicted in Figure 2, we select parents in pairs to produce two new offspring. We select first two random genes: the first one in the range [1 to half] and the second one from [half + 1 to last] of the chromosome. In this manner, we try to maintain a crossover of both the control and parameter genes in the chromosome. For the mutation process, we mutate all offspring from crossover. This comprises a percentage of genes from each children chromosome, and depending on the position, we can mutate it in two ways:

- Flip-bit mutation: if the chosen gene is a control gene, we invert its value (0 goes to 1, and 1 goes to 0).
- Random mutation: if the chosen gene is a parameter gene, we change its value with a new value randomly generated from the range from 1 to 4.

The new population (the population that passes to the next generation) is generated from an elitist selection from the parents and its offspring, where they are ranked in a list from the best to the worst, and we select only the best individuals to create the new population with the same size as the previous one. With this, all bad solutions are deleted and do not matter if it is a parent or an offspring, to ensure that only the best individual passes to the next generation.

### 3.4. The Knapsack Problem

The knapsack problem is about, given a set of items, getting a subset of them, so it satisfies the constraints described in equations (3) and (4), where *n* is the number of items on the set, *p*_*i*_ is the profit of item *i*, *x*_*i*_ is the number of copies (0 or 1) of item *i*, *w*_*i*_ is the weight of item *i*, and *W* is the maximum weight limit of the knapsack. The weight from the subset of items needs to be less or equal to the maximum weight limit and also be the subset that maximizes the profit:

(3) $$\begin{array}{c} \max\sum_{i=1}^{n}p_{i}x_{i}, \end{array}$$

(4) $$\begin{array}{c} \text{subject to }\sum_{i=1}^{n}w_{i}x_{i}\leq W\text{ and }x_{i}\in \left\{0,1\right\}. \end{array}$$

Eight hundred randomly generated instances create the testbed used for this work. We used the generator proposed by Pisinger [43]) to produce such instances. From these instances, there are 200 instances suitable for each of the heuristics. The former means that we generated instances until we have 200 instances where just one heuristic is better than the others (this was done to create a testbed of instances that is balanced, and no heuristic has any advantage over the others on the whole set). Each instance contains 40 items with a weight ranging from 1 to 32 and a profit ranging from 1 to 128, with a maximum capacity of the knapsack for all instances of 25. To observe the advantages in learning from our proposed fuzzy approach against the selection hyperheuristics, we selected 30 instances from each heuristic for training. So, from all these 800 instances, we randomly selected 15% (or 30 instances for each heuristic) for training and the remaining 85% for testing, having 120 instances for training and 680 instances for testing. We created both sets of instances in a way that each one is still a balanced set. This is, we want to prove that, with a few instances, our fuzzy hyperheuristic can learn the process of solving a knapsack problem and test it with a broader set of instances. We are also using the hard instances proposed by Pisinger [8] as a test set, which corresponds to the instances with 20, 50, 100, and 200 items and around 1000 as a maximum capacity of the knapsack. We include 600 instances for each one of these types, producing in total 2400 instances.

### 3.5. The Features

A hyperheuristic has to decide which heuristic to use at a given decision point. For this reason, it is very important to characterize the problem state by using the appropriate set of features. To picture the current state of an instance (while being solved), we work with seven features based on the list of remaining items that have not been packed yet. Then, every time an item is removed from the list, the features are recomputed. We compute some features based only on the weight of the items and others only on their profit. These features can give us a view of the state of the problem and help to determine the selection of the next heuristic. We give next a brief description of each feature:

- **MeanW** is the average value of the weights from all remaining items.
- **MedianW** is the median value of the weights from all remaining items.
- **StdW** is the standard deviation of the weights from all remaining items.
- **MeanP** is the average value of the profits from all remaining items.
- **MedianP** is the median value of the profits from all remaining items.
- **StdP** is the standard deviation of the profits from all remaining items.
- **Corr** is the correlation value between the weights and the profits.

### 3.6. The Heuristics

To solve the knapsack problem, we chose the next four heuristics from the literature which are the most commonly used in this problem [44, 45]. These heuristics dictate which item should be selected from the list of items to be packed. These heuristics select the next item based on the following criteria:

- **Default:** it selects the first item in the list.
- **MaxP**: it selects the item with the maximum profit value from the list.
- **MinW**: it selects the item with the minimum weight value from the list.
- **MaxPW**: it selects the item with the maximum value for the quotient profit over weight calculation.

After defining the heuristics and the features of a problem, the general algorithm for the selection hyperheuristics works as follows. First, from an instance, we compute all features. In the hyperheuristic, it computes a distance from all rules to the current features and decides which heuristic applies. After it applies the heuristic, the problem state changes, so all features are computed again to measure the distance for the selection of the next heuristic, until the problem is solved or it meets termination criteria. Hyperheuristics (and our fuzzy approach) can use up to seven features to decide which of the four heuristics are selected at a moment of the execution of the algorithm. To accomplish this task, the metaheuristic PSO is used to optimize the set of rules from the hyperheuristics and the GA for the set of fuzzy rules from the FIS.

## 4. Methodology and Solution Model

**Figure 3:**
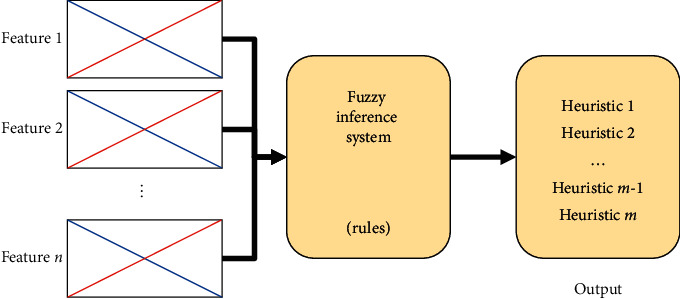
Fuzzy inference system used as hyperheuristic. It can have *n* features as inputs 2^*n*^ rules and one output with *m* heuristics.

We use the fuzzy inference system depicted in Figure 3 as a base model for our fuzzy approach. This FIS is Sugeno type and can have up to *n* features and *m* heuristics. In this paper, we use up to seven features and four heuristics, which are the inputs and the output of the fuzzy inference system. The number of fuzzy rules in the FIS is given by the number of inputs and membership functions for each of these inputs. The membership functions of the inputs of the FIS depicted in Figure 3 are triangular because, from the types of membership functions, these are the easiest to manipulate. All inputs have two triangular membership functions: “low” and high” described by equation (5). The output represents the available heuristics, which are represented by constant output membership functions:

- **Parameters for “low”**: *a*=−1, *b*=1, *m*=0.
- **Parameters for “high”**: *a*=0, *b*=2, *m*=1.

(5) $$\begin{array}{c} \mu_{A}\left(x\right)=\left\{\begin{array}{cc} 0, & x\leq a,\\ \frac{x-a}{m-a}, & a<x\leq m,\\ \frac{b-x}{b-m}, & m<x<b,\\ 0, & x\geq b. \end{array}\right. \end{array}$$

To search for the consequent of each fuzzy rule, the GA must use the chromosome, as illustrated in Figure 1, with a size of 256 genes for all the fuzzy rules, where it includes control genes and parameter genes. Here, control genes show which rules are considered in the system and which ones are ignored, using a procedure for activation or deactivation of fuzzy rules using the values 1 or 0, respectively. Parameter genes show the heuristic recommended by each rule (values ranging from 1 to 4). The decoding process is as follows:

- Take an individual from the population.
- Each consequent (from the parameter dimensions) is assigned into its corresponding rule.
- With the values from the control dimensions, the rules will be deleted if 0 or kept if 1.
- The remaining set of fuzzy rules is saved into the fuzzy hyperheuristic (depicted in Figure 3) and is ready to be applied to solve the instances of the knapsack problem using Algorithm 1.

The GA must convert a chromosome into a fuzzy rule set to be included and evaluated as a fuzzy hyperheuristic. The GA uses the solving process from Algorithm 1 to test an individual and compute its fitness using the training set. We can apply the same process to solve another set of instances like testing or other knapsack problems. It is important to mention that the fuzzy hyperheuristic used in Algorithm 1 can be an optimized FIS or a FIS that is being optimized; that is, the process described in Algorithm 1 represents the way in which we compute the fitness from an individual of the population of the GA. While a fuzzy inference system is being optimized by GA, an individual is transformed into a set of fuzzy rules and integrated into the FIS for solving the training set and get its fitness. On the contrary, an optimized FIS can be applied to solve the testing set of instances to see its performance once optimized. The evaluation process depicted in Algorithm 1 illustrates how a fuzzy hyperheuristic solves a set of instances.

The rules for the hyperheuristics are coded in the following manner. Taking the example rules from equation (1), in our case, each rule has 7 features and 1 last element to indicate the heuristic per rule, which means that each rule has a length of 8. So, PSO will need a chromosome of 8 × 4=32 dimensions to optimize the rules of the hyperheuristic with 4 rules, 8 × 6=48 dimensions for the 6-rule hyperheuristic, and 8 × 8=64 dimensions for the 8-rule hyperheuristic.

## 5. Experiments and Results

The parameters used to run the GA and PSO are described as follows. Both algorithms run for 100 iterations by using a population of 30 individuals. For the GA, we used 256 genes and 0.8 and 0.1 as crossover and mutation rates, respectively. For PSO, we use 32, 48, and 64 dimensions to find the rules for the selection hyperheuristics with 4, 6, and 8 rules, respectively. The inertia weight is linear, decreasing from 0.9 to 0.1 over the iterations. Also, as suggested by Rashedi [46], the values of *C*_1_ and *C*_2_ were both set to 2. We use these two metaheuristics for the optimization of the rules in our hyperheuristics. The difference is that PSO is used to generate the traditional selection hyperheuristics, and the GA generates the fuzzy rules of the FIS in our fuzzy approach. In the end, we compare the selection hyperheuristics optimized by PSO against the fuzzy hyperheuristics optimized by GA. The optimization process held by PSO for the selection hyperheuristics was in the following manner. Each particle represents a set of rules and its corresponding heuristic. The number of features is the size of each rule plus a value for the heuristic. The decodification process converts each particle into a set of rules by dividing it onto a set of vectors representing the rules. The last item in the vector is the heuristic. In this way, PSO can find the corresponding values for each rule from each heuristic.

For comparison, we apply a binary genetic algorithm to solve directly the knapsack problem, to have a point of view from a metaheuristic perspective. So, in this case, we are using the same algorithm (but different parameters and configuration of the chromosomes) as the GA used to optimize the consequents of the fuzzy rules from our fuzzy hyperheuristic approach, but now, we are using chromosomes that only have genes with the value 0 or 1. Also, a chromosome has the same length as the number of items per instance; with this, if the *n* gene has a value of 1, then the *n* item will be added to the knapsack, or not if this value is 0. The main difference between this metaheuristic and the hyperheuristic is that GA will not save any knowledge of the problem, and in every instance, a new population needs to be evolved to bring the best solution to the instance. The parameters used to run this version of GA are as follows: the algorithm will run for 10 generations with a population of 20 chromosomes and 0.80 and 0.05 as crossover and mutation rates, respectively. For the size of the chromosome, this will vary depending on the number of items in an instance; in our case, the numbers of items are 20, 40, 50, 100, and 200.

We use a synthetic hyperheuristic called Oracle (which represents the best heuristic for each instance). The Oracle works by using all heuristics to solve an instance, and the result of the best heuristic is saved as the result of the Oracle method. In other words, the Oracle is a hyperheuristic which always select the best heuristic but only after each heuristic is applied to solve the problem. The results from the heuristics and the Oracle only bring one result. If we solve the same set of instances with the same heuristic or the Oracle, we will get the same result. However, for each type of selection hyperheuristic or the fuzzy approach, we generate 30 of them. So, in the case of hyperheuristics, we can bring the average, standard deviation, and best and worst results from these 30 variations and applied to solve the instances from the testing set.  Table 1 shows these results. Note that there are 680 instances in the testing set and in the standard deviation metric, and we include the coefficient of variation as a percentage: (*σ*/*μ∗*100%).

Table 1 shows that, after the Oracle, our proposed fuzzy hyperheuristic approach gets the best results on average, standard deviation, and best and worst metrics of the testing set. The results from the binary GA approach are bad when compared to the hyperheuristics, but with more generations, this approach can overcome the other methods. The only disadvantage is that, on every instance, the binary GA needs to evolve an entire population to find a solution, and the hyperheuristics and the fuzzy approach only need to see the instances for training to be able to solve the unseen instances from the testing set.  Table 1 shows that traditional selection hyperheuristics get better results than the heuristics but worst than the fuzzy approach. The reason why the hyperheuristics and the fuzzy approach are better than the heuristics is because the heuristics are simple methods which are faster but does not always guarantee the global best. On the contrary, hyperheuristics create a symphony of heuristics and are driven by the features of the problem, and when the features change, the hyperheuristics select the best heuristic for the current features. The Oracle synthetic hyperheuristic is our goal in the developing of any kind of hyperheuristics. The results presented in Table 1 show that the Oracle gets the best results, and this is because it always selects the best heuristic for each instance. However, there are some instances were the hyperheuristics (fuzzy approach included) get better or equal on results of profit than the Oracle method. These results are presented in Table 2, where the percentages are computed based on the total of instances solved between all instances of the testing set: Percentage=(Number_of_instances/680^*∗*^100%). From the results in Table 2, we can observe that the fuzzy approach gets the best results in the majority of the metrics used, except in the standard deviation. These results show that the fuzzy hyperheuristic gets better or equal results than the Oracle in 44.87% on average of the total instances solved. It is important to remember that we generate all instances from the sets for training and testing randomly. For each instance, one heuristic, in particular, was better than the others. This means that each heuristic is the best in 25% of the instances, but the Oracle knows which is the best one. With this in mind, it is clear why the Oracle gets the best results. This is why we need another set of instances for testing the already optimized methods.

All methods are used to solve a series of instances from Pisinger [8], called “hard instances,” where these are only for testing purpose. We tested all trained methods without modification or further optimization (except for the binary GA method which for each instance needs to evolve a population to find the solution).  Table 3 presents the results from all methods applied to the 2400 hard instances for testing, where it shows the results on profit got, in terms of average, standard deviation, and best and worst results from each of the 30 different hyperheuristics of each model. In this table, we are also including the coefficient of variation as a percentage in the standard deviation of the results, to see the differences between the fuzzy approach and the traditional selection hyperheuristics. We are using four types of hard instances with 20, 50, 100, and 200 items, with 600 instances per each set, giving 2400 hard instances for testing all methods. Results from the hard instances illustrated in Table 3 show the behavior of the exact methods, where the heuristic ”Default” gets better results than “MaxP” in less time. The heuristic “MaxPW” is still the best heuristic. The Oracle hyperheuristic gets the best results. Results of Table 3 show that the proposed fuzzy hyperheuristic gets better results on average and the lowest standard deviation values, when compared with the three selection hyperheuristic models. It also shows that the binary GA method got a better result on average than our proposed approach in the instances with 20 items and got the best results on standard deviation in all the instances. Nevertheless, Table 3 shows that the selection hyperheuristic with 4 rules (HH4) gets the best individual results on the set of instances with 50, 100, and 200 items. The coefficient of variation also shows that the fuzzy approach is more stable in its results because the standard deviation is the lowest, and the results are the best on average than the other hyperheuristics. Even though the binary GA got the lowest values on standard deviation, its results on average are poor, except for the results from the instances with 20 items, where it got an excellent result on average.

Table 4 shows the percentage of how many times the trained hyperheuristics get better or equal results on the 600 instances per each set. From these results, we can see that the fuzzy approach has an advantage than the traditional selection hyperheuristics. Nevertheless, the selection hyperheuristics get great results, but compared with the fuzzy approach, it is clear that the inclusion of a fuzzy inference system in the inner working of a selection hyperheuristic is beneficial and helps in getting better quality results. All results from Table 4 are computed using percentage=(number_of_instances/600^*∗*^100%).

The proposed fuzzy approach has the particularity that, after being optimized, it will select mostly two of the heuristics (MaxP and MaxPW), and depending on the problem, it has the ability to switch between them. This behavior is important since selection hyperheuristics most times select the best heuristic.  Table 5 contains the percentage of selection of heuristics on average by each 30 hyperheuristics, when applied to each set of instances. We also show the total selected heuristics by each method per set of instances and which is the most used heuristic based on the percentage of use. There are many instances of the knapsack problem, in which the selection hyperheuristics only select the best heuristic which is MaxPW. In fact, results from Table 5 show that, on average, selection hyperheuristics follow the next pattern where MaxPW is the most selected, followed by MinW and MaxP, and the less selected is default. Also, it does not matter if the problem has different features, like when we change from the set for testing to the hard instances. The traditional selection hyperheuristics still follow the same pattern. From the results of the total selected heuristics, all the selection hyperheuristics make a selection of 816000 heuristics in the set of instances for testing. It is based on the fact that there are a total of 816000 items in the set of instances for testing (680 instances of 40 items each for 30 hyperheuristics). We assume that each of the traditional selection hyperheuristics work with all items before the knapsack is full, but the fuzzy approach only selects 572522 heuristics which means that some knapsacks are full before it reaches all items in the instance. All this tells us that the fuzzy approach makes better decisions on the selected heuristics because it needs to select less heuristics in the process of solving an instance. On the contrary, the fuzzy approach selects Default and MinW just a few times, and in the set for testing, MaxP is the most selected followed by MaxPW, but in the hard instances, it changes, and MaxPW is now the most selected followed by MaxP. We believe that this behavior is due to the way in which a fuzzy inference system takes the features as inputs and transforms them by means of fuzzy sets in degrees of membership, to later unite all the firing strengths of the fuzzy rules and at the end brings an output through the defuzzification process.

We finally present the best hyperheuristics based on the results from the testing set of instances. The rules are presented in the form of a matrix. These rules are the result of the optimization process held by PSO. The rules of the hyperheuristics are presented in equation (6) for the best 4-rule hyperheuristic, equation (7) for the best 6-rule hyperheuristic, and equation (8) for the best 8-rule hyperheuristic. The rules of the best FIS in the fuzzy approach are presented in equation (9). These rules are the result of the optimization of GA from the total of 128 rules from the beginning. The GA optimizes the number of rules and tries to find the best consequent for each fuzzy rule. The rules from the fuzzy inference system in equation (9) were optimized by the GA. There are a total of 15 fuzzy rules using only 5 features which are as follows: the mean, median, and standard deviation of the weights, the median of the profits, and the correlation between the weights and the profits from all the remaining items in the instance:

(6) $$\begin{array}{c} \text{HH}4_{\text{Rules}}=\left[\begin{array}{cccccccc} 0.3050 & 0.8297 & 0.8401 & 0.4919 & 0.6046 & 0.6499 & 0.9743 & 3\\ 0.8561 & 0.8327 & 0.4889 & 0.1796 & 0.6799 & 0.8800 & 0.8456 & 1\\ 0.9148 & 0.3480 & 0.0682 & 0.5154 & 0.3796 & 0.7554 & 0.5197 & 2\\ 0.2230 & 0.4073 & 0.1687 & 0.6771 & 0.5526 & 0.6501 & 0.2346 & 4 \end{array}\right], \end{array}$$

(7) $$\begin{array}{c} \text{HH}6_{\text{Rules}}=\left[\begin{array}{cccccccc} 0.1445 & 0.5143 & 0.1327 & 0.5787 & 0.5424 & 0.5878 & 0.6423 & 4\\ 0.1077 & 0.7055 & 0.7022 & 0.2693 & 0.1643 & 0.4226 & 0.4865 & 2\\ 0.0877 & 0.4774 & 0.5443 & 0.1795 & 0.5553 & 0.4256 & 0.2052 & 4\\ 0.7413 & 0.5267 & 0.3172 & 0.1156 & 0.7187 & 0.4712 & 0.8156 & 2\\ 0.4322 & 0.1651 & 0.1941 & 0.3388 & 0.8479 & 0.8611 & 0.9912 & 3\\ 0.4774 & 0.0277 & 0.6285 & 0.7475 & 0.0820 & 0.4760 & 0.1824 & 3 \end{array}\right], \end{array}$$

(8) $$\begin{array}{c} \text{HH}8_{\text{Rules}}=\left[\begin{array}{cccccccc} 0.6730 & 0.8060 & 0.7614 & 0.4829 & 0.4522 & 0.0549 & 0.5093 & 3\\ 0.1832 & 0.6296 & 0.4090 & 0.4644 & 0.6313 & 0.9099 & 0.4093 & 3\\ 0.0482 & 0.5832 & 0.3192 & 0.1975 & 0.5213 & 0.1740 & 0.1743 & 3\\ 0.3065 & 0.4228 & 0.9555 & 0.2742 & 0.3936 & 0.2426 & 0.1143 & 1\\ 0.8092 & 0.4996 & 0.1078 & 0.6202 & 0.3630 & 0.1916 & 0.5458 & 2\\ 0.4771 & 0.5313 & 0.5219 & 0.4525 & 0.4035 & 0.4390 & 0.3831 & 4\\ 0.8770 & 0.7844 & 0.3277 & 0.4401 & 0.4666 & 0.6499 & 0.2835 & 4\\ 0.3411 & 0.1472 & 0.7087 & 0.2996 & 0.7242 & 0.4152 & 0.3837 & 1 \end{array}\right], \end{array}$$

(9) $$\begin{array}{c} {\text{Fuzzy}}_{\text{Rules}}=\left[\begin{array}{cccccc} \text{Low} & \text{Low} & \text{Low} & \text{High} & \text{Low} & 4\\ \text{Low} & \text{Low} & \text{High} & \text{Low} & \text{Low} & 4\\ \text{Low} & \text{Low} & \text{High} & \text{High} & \text{High} & 4\\ \text{Low} & \text{High} & \text{Low} & \text{Low} & \text{Low} & 2\\ \text{Low} & \text{High} & \text{Low} & \text{Low} & \text{High} & 3\\ \text{Low} & \text{High} & \text{Low} & \text{High} & \text{High} & 4\\ \text{Low} & \text{High} & \text{High} & \text{Low} & \text{Low} & 4\\ \text{High} & \text{Low} & \text{Low} & \text{Low} & \text{High} & 1\\ \text{High} & \text{Low} & \text{Low} & \text{High} & \text{High} & 1\\ \text{High} & \text{Low} & \text{High} & \text{Low} & \text{Low} & 3\\ \text{High} & \text{Low} & \text{High} & \text{High} & \text{Low} & 4\\ \text{High} & \text{High} & \text{Low} & \text{Low} & \text{Low} & 2\\ \text{High} & \text{High} & \text{High} & \text{Low} & \text{Low} & 3\\ \text{High} & \text{High} & \text{High} & \text{Low} & \text{High} & 4\\ \text{High} & \text{High} & \text{High} & \text{High} & \text{Low} & 3 \end{array}\right]. \end{array}$$

## 6. Discussion of Results and Statistical

**Figure 4:**
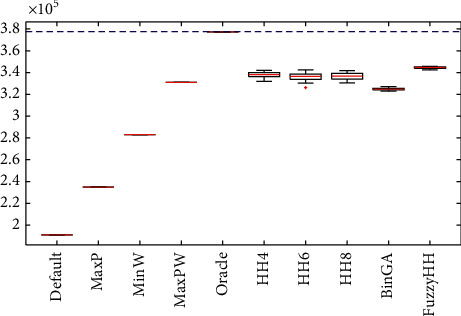
Results from all methods applied to solve the set of 680 instances for testing. Boxplot of the results.

**Figure 5:**
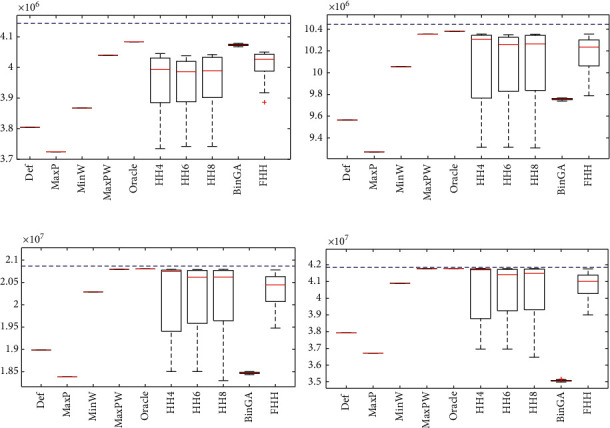
Results from all methods using the set of hard instances for testing. Boxplot of the results with the testing set of hard instances. (a) Hard20. (b) Hard50. (c) Hard100. (d) Hard200.

**Figure 6:**
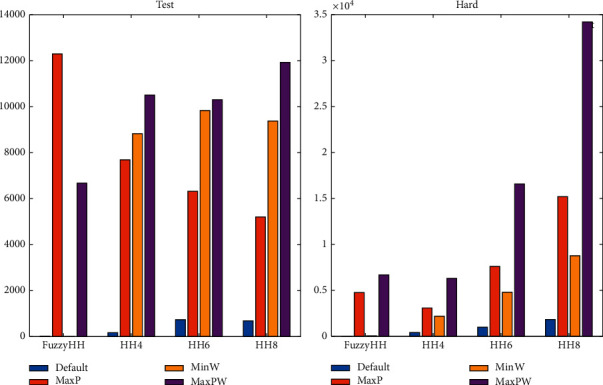
Number of heuristics selected per method on all sets (how many times a method selects a heuristic on each set of instances).

It is hard to see results in its full potential with just a table full of values, so in this section, we will present the results in a graphical view to see the differences in a better way. It is important to mention that we compute the optimal value from all instances using dynamic programming (marked as a dashed blue line in the figures), but because of its humongous computational time, it is not worth using it.  Figure 4 shows an improvement from a selection hyperheuristic (HH4, HH6, and HH8) versus a fuzzy hyperheuristic, where all results from our fuzzy approach are better than any results from selection hyperheuristics. We illustrate the results with the four sets of hard instances using all methods in Figure 5, where it is shown the performance of hyperheuristics in a set of instances is very different from those used for training and testing. Also, it shows that our fuzzy approach gets better results when compared with the selection hyperheuristics. From all results in Figure 5, we observe that the heuristic MaxP changes its performance and now is worse than the other heuristics, while MaxPW is sometimes better than hyperheuristics. However, the proposed fuzzy hyperheuristics gets more closed results (with less standard deviation) than the selection hyperheuristics.  Figure 6 illustrates how many times a method selects each heuristic on average in all sets of instances, using the selection hyperheuristics and the fuzzy hyperheuristics. The results from Figure 6 show that our proposed approach changes the percentages of the heuristics selected. While on set of instances for testing, it selects mostly MaxP followed by MaxPW; now in the hard instances, it changes to select more MaxPW followed by the MaxP. From the hard instances, the difference between the Oracle and the best heuristic is tiny, so there is no hyperheuristic that can get better results than the best heuristic.

When analyzing the results, we found variations in the process to train a given method, so this is the reason we ran some statistical tests to determine if there was a better method in terms of average and variance. No variations were found in the results for the exact methods, the trained hyperheuristic and the fuzzy hyperheuristic. We have at least 30 results for each tested method and because each of these results are an independent run with the same parameter, we perform a statistical *Z*-test to know if our proposed fuzzy hyperheuristic is better than a selection hyperheuristic approach. This test will address the different sets of instances used and the different metaheuristics used to optimize the methods. The parameters of the statistical *Z*-test are as follows: a significance level of 5%, a critical value of 1.645, *μ*_1_ is our fuzzy approach, *μ*_2_ is any other method, the null hypothesis (*H*_0_) is *μ*_1_ ≤ *μ*_2_, and the alternative hypothesis (*H*_*a*_) is *μ*_1_ > *μ*_2_. Null hypothesis (*H*_0_) says that our proposed method gets on average less or equal (worst) results than the other method, and the alternative hypothesis (*H*_*a*_) state that our fuzzy approach gets on average greater (better) results than the other method. We present the results of the statistical test in Table 6, where it shows a comparison with the fuzzy approach and all the other hyperheuristics. The results from Table 6 show that (9/20) results cannot reject the null hypothesis. For the results above the critical value of 1.645, there is enough evidence to reject the null hypothesis. Therefore, we can say that our proposed fuzzy hyperheuristic gets on average better results than all selection hyperheuristics in the training and testing sets, but in the sets of hard instances, it only gets on average better results than all the selection hyperheuristics in the set with 20 items. In the comparison against the binary GA, there is enough evidence to say that our proposed approach gets better results in almost all sets, except for the hard instances with 20 items. We need to mention that the binary GA approach with enough generations will get better results than our proposed approach, but it will not save any knowledge, and it does not need a training phase because it always needs to evolve a population for every instance.

## 7. Conclusions

Hyperheuristics get better results than the heuristics alone. Our proposed fuzzy hyperheuristic approach helps in getting better quality results when is included in the inner working of a selection hyperheuristic. The fuzzy approach gets better results with an increase in computational time, slower than a selection hyperheuristic with 4 rules, but at the same level than the others with 6 or 8 rules. The proposed approach gets better results in a controlled environment (with the sets of instances for training and testing), and when it is applied to different instances (the hard instances), it helps to improve the quality of the results. But if the results from the best heuristic are closer to the results from the Oracle, then it is a problem where no hyperheuristic can improve the quality of the results (like the hard instances) and even with that our proposed approach gets better quality results than the selection hyperheuristics in the hard instances with 20 items. Statistical test shows that there is enough evidence to say that our proposed fuzzy hyperheuristic gets better results in half of the sets of instances than selection hyperheuristics (also when compared with heuristics since hyperheuristics get better results), but in the three more difficult problems, we cannot get enough evidence because of the closeness in the results from the best heuristic and the Oracle. Against the binary GA method, our approach got better results in 4/5 sets of instances, even when the binary GA evolves a population of chromosomes for each instance, while our proposed approach only sees the instances from the training set. Based on the results of the total selected heuristics, we can state that our fuzzy approach made better decisions because it used fewer heuristics than the traditional selection hyperheuristics. In fact, the fuzzy approach uses 22% fewer heuristics than the hyperheuristic with four rules, 46% fewer than the hyperheuristic with six rules, and 65% fewer heuristics than the hyperheuristic with eight rules. This means that the fuzzy approach requires fewer operations in an instance to fulfill a knapsack and gets the best profit from it. Future work includes the application of our proposed approach into different problem domains to see the performance and differences between these and the selection hyperheuristics. Also in another direction for future work, we can use more advanced techniques to find better features or combine them to help the fuzzy inference system select a heuristic.

## Figures and Tables

**Algorithm 1:**
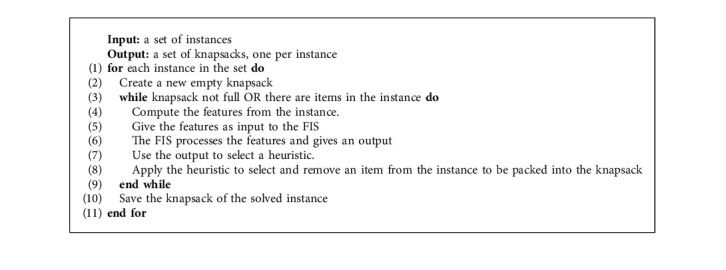
Solving a set of instances.

**Table 1:** Results in profit from all methods in the testing set.

Methods	Metrics			
	Average	Standard deviation	Best	Worst
**Default**	191080	0	191080	191080
**MaxP**	234917	0	234917	234917
**MinW**	282972	0	282972	282972
**MaxPW**	330949	0	330949	330949
**Oracle**	**377244**	**0**	**377244**	**377244**
**HH4**	337979.36	2543.48 (0.75%)	342036	331936
**HH6**	336062.20	3369.03 (1.00%)	342381	326238
**HH8**	336574.20	2918.92 (0.86%)	341739	330495
**Binary GA**	324800.33	930.31 (0.28%)	326982	323036
**FuzzyHH**	**344353.66**	759.35 (0.22%)	**345661**	**342531**

Results in bold are the best.

**Table 2:** Percentage of times when the hyperheuristics get better or equal results than the Oracle applied to the testing set of instances.

Metrics		Methods			
		HH4	HH6	HH8	FuzzyHH
**Average**	**Equal**	41.10%	39.24%	39.26%	**44.67%**
	**Better**	0.12%	0.05%	0.06%	**0.20%**

Standard deviation	**Equal**	5.13%	4.41%	5.34%	**1.97%**
	**Better**	0.19%	**0.11%**	0.13%	0.13%

**Best**	**Equal**	**47.94%**	45.58%	47.20%	**47.94%**
	**Better**	0.44%	0.44%	0.44%	**0.58%**

**Worst**	**Equal**	28.23%	29.55%	25.88%	**41.32%**
	**Better**	0	0	0	0

Percentage=(number_of_instances/680^*∗*^100%). Results in bold are the best.

**Table 3:** Results from the hyperheuristics with the 2400 hard instances.

**Methods**		**Hard Pisinger instances**			
		**20**	**50**	**100**	**200**
**Default**		3804271	9562959	18985439	37922860
**MaxP**		3724588	9269841	18382930	36723430
**MinW**		3867345	10055148	20287616	40898138
**MaxPW**		4039708	10353555	20791627	41776413
**Oracle**		**4084003**	**10380851**	**20802281**	**41783753**
**HH4**	**Average**	3949205.76	10048476.70	20107840.33	40333536.33
	Standard deviation	99734.35 (2.52%)	380935.64 (3.79%)	874132.26 (4.34%)	1871087.96 (4.63%)
	**Best**	4045507	**10355658**	**20791627**	**41776413**
	**Worst**	3734571	9312890	18502165	36963737

**HH6**	**Average**	3950021.50	10079568.63	20193352.10	40525684.03
	Standard deviation	84499.05 (2.13%)	324209.91 (3.21%)	747259.32 (3.70%)	1601814.81 (3.95%)
	**Best**	4038167	10350020	20786768	41775010
	**Worst**	3741617	9314130	18502041	36957315

**HH8**	**Average**	3957540.76	10089769.23	20192245.86	40509777.40
	Standard deviation	92317.06 (2.33%)	333676.40 (3.30%)	777075.35 (3.84%)	1681688.34 (4.15%)
	**Best**	4042198	10353705	**20791627**	**41776413**
	**Worst**	3741978	9307530	18303016	36466476

**Binary GA**	**Average**	**4073227.76**	9755928.90	18475255.43	35069085.23
	Standard deviation	3096.26 (0.07%)	7888.44 (0.08%)	17760.93 (0.09%)	41037.09 (0.11%)
	**Best**	**4078723**	9767818	18503545	35167446
	**Worst**	**4067968**	9738018	18433895	34989419

**FuzzyHH**	**Average**	**4008457.76**	**10175892.16**	**20342907.40**	**40794413.63**
	Standard deviation	43384.17 (1.08%)	155492.79 (1.52%)	359855.26 (1.76%)	772999.81 (1.89%)
	**Best**	**4050373**	10355610	20777933	41757401
	**Worst**	**3886225**	**9787082**	**19477163**	**39003392**

Results in bold are the best.

**Table 4:** Percentage of times when the hyperheuristics get better or equal results than the Oracle applied to the sets of hard instances.

**Metrics**		**Set of instances**	**Methods**			
			**HH4**	**HH6**	**HH8**	**FuzzyHH**
**Average**	**Equal**	**20**	43.67%	51.75%	56.14%	**59.94%**
		**50**	41.31%	50.55%	55.10%	**59.13%**
		**100**	43.01%	51.38%	55.00%	**59.08%**
		**200**	**38.64%**	36.95%	35.26%	33.90%
	**Better**	**20**	3.10%	3.30%	2.76%	**7.13%**
		**50**	2.47%	2.60%	1.97%	**5.18%**
		**100**	1.74%	1.69%	1.27%	**3.22%**
		**200**	0.82%	0.96%	0.66%	**1.73%**

Standard deviation	**Equal**	**20**	12.19%	12.19%	13.12%	**8.05%**
		**50**	19.67%	18.40%	20.39%	**10.63%**
		**100**	25.03%	22.80%	25.76%	**14.38%**
		**200**	29.45%	26.67%	30.12%	**17.16%**
	**Better**	**20**	3.96%	3.72%	3.44%	**2.76%**
		**50**	3.11%	3.05%	**2.46%**	2.72%
		**100**	2.06%	1.86%	**1.63%**	1.86%
		**200**	0.92%	0.87%	**0.73%**	1.37%

**Best**	**Equal**	**20**	59.83%	74.83%	83.83%	**93.16%**
		**50**	59.33%	74.16%	83.16%	**92.83%**
		**100**	59.66%	74.83%	83.83%	**93.16%**
		**200**	55.16%	66.16%	74.33%	**82.33%**
	**Better**	**20**	12.50%	12.16%	12.50%	**13.66%**
		**50**	9.16%	9.00%	8.00%	**12.00%**
		**100**	**7.16%**	6.16%	6.33%	6.16%
		**200**	3.16%	2.50%	2.66%	**5.16%**

**Worst**	**Equal**	**20**	21.00%	14.16%	20.00%	**28.50%**
		**50**	16.83%	12.66%	13.83%	**23.50%**
		**100**	15.33%	12.16%	9.50%	**17.66%**
		**200**	13.83%	11.66%	7.00%	**14.33%**
	**Better**	**20**	0	0	0	**2.00%**
		**50**	0	0	0	**0.66%**
		**100**	0	0	0	**0.50%**
		**200**	0	0	0	**0.16%**

Results in bold are the best.

**Table 5:** Heuristic selection percentage per method on each set of instances.

**Methods**	Set of instances	**Heuristics**				Total selected heuristic	Most used heuristic
		**Default** (%)	**MaxP** (%)	**MinW** (%)	**MaxPW** (%)		
**HH4**	**Test**	0.62	28.27	32.47	38.64	816000	MaxPW
	**Hard**	3.05	25.34	14.48	57.13	360000	MaxPW

**HH6**	**Test**	2.70	23.25	**36.17**	37.88	816000	MaxPW
	**Hard**	3.37	21.18	19.11	56.34	900000	MaxPW

**HH8**	**Test**	2.52	19.15	34.48	43.85	816000	MaxPW
	**Hard**	**5.26**	19.44	16.63	**58.67**	1800000	MaxPW

**FuzzyHH**	**Test**	0.12	**64.71**	0.02	35.15	**572522**	MaxP
	**Hard**	0.05	44.31	0.42	55.22	**340324**	MaxPW

Results in bold indicate the highest percentage of use of each heuristic.

**Table 6:** Results of the statistical test.

**Other methods (** *μ*_2_ **)**	**Set of instances**	**FuzzyHH (** *μ*_1_ **)**
**HH4**	**Test**	**12.2448**
	**Hard20**	**2.9839**
	**Hard50**	**1.6961**
	**Hard100**	1.3620
	**Hard200**	1.2469

**HH6**	**Test**	**12.5431**
	**Hard20**	**3.3696**
	**Hard50**	1.4672
	**Hard100**	0.9876
	**Hard200**	0.8275

**HH8**	**Test**	**13.3465**
	**Hard20**	**2.7340**
	**Hard50**	1.2813
	**Hard100**	0.9636
	**Hard200**	0.8423

**Binary GA**	**Test**	**9.1834**
	**Hard20**	−8.1564
	**Hard50**	**4.7742**
	**Hard100**	**4.3923**
	**Hard200**	**6.5108**

Results in bold are when the test rejects the null hypothesis.

## Data Availability

The knapsack instances data supporting this research are from previously reported studies and datasets, which have been cited. The processed data are available at http://hjemmesider.diku.dk/∼pisinger/codes.html.
